# Frequency of CXCR3^+^ CD8^+^ T-Lymphocyte Subsets in Peripheral Blood Is Associated With the Risk of Paradoxical Tuberculosis-Associated Immune Reconstitution Inflammatory Syndrome Development in Advanced HIV Disease

**DOI:** 10.3389/fimmu.2022.873985

**Published:** 2022-04-01

**Authors:** Rafael Tibúrcio, Gopalan Narendran, Beatriz Barreto-Duarte, Artur T. L. Queiroz, Mariana Araújo-Pereira, Selvaraj Anbalagan, Kaustuv Nayak, Narayanan Ravichandran, Rajasekaran Subramani, Lis R. V. Antonelli, Kumar Satagopan, Komathi Anbalagan, Brian O. Porter, Alan Sher, Soumya Swaminathan, Irini Sereti, Bruno B. Andrade

**Affiliations:** ^1^ Laboratório de Inflamação e Biomarcadores, Instituto Gonçalo Moniz, Fundação Oswaldo Cruz, Salvador, Brazil; ^2^ Multinational Organization Network Sponsoring Translational and Epidemiological Research (MONSTER) Initiative, Salvador, Brazil; ^3^ Faculdade de Medicina, Universidade Federal da Bahia, Salvador, Brazil; ^4^ Department of Clinical Research, National Institute for Research in Tuberculosis, Chennai, India; ^5^ Curso de Medicina, Universidade Salvador (UNIFACS), Salvador, Brazil; ^6^ Programa de Pós-Graduação em Clínica Médica, Universidade Federal do Rio de Janeiro, Rio de Janeiro, Brazil; ^7^ Center of Data and Knowledge Integration for Health (CIDACS), Instituto Gonçalo Moniz, Fundação Oswaldo Cruz, Salvador, Brazil; ^8^ ICGEB-Emory Vaccine Centre, International Centre for Genetic Engineering and Biotechnology, New Delhi, India; ^9^ Government Hospital of Thoracic Medicine, Chennai, India; ^10^ Laboratório de Biologia e Imunologia de Doenças Infecciosas e Parasitárias, Instituto René Rachou, Fundação Oswaldo Cruz, Belo Horizonte, Brazil; ^11^ HIV Pathogenesis Section, Laboratory of Immunoregulation, National Institute of Allergy and Infectious Diseases, National Institutes of Health, Bethesda, MD, United States; ^12^ Immunobiology Section, Laboratory of Parasitic Diseases, National Institute of Allergy and Infectious Diseases, National Institutes of Health, Bethesda, MD, United States; ^13^ Wellcome Trust Centre for Infectious Disease Research in Africa, Institute of Infectious Disease and Molecular Medicine, University of Cape Town, Cape Town, South Africa; ^14^ Curso de Medicina, Escola Bahiana de Medicina e Saúde Pública (EBMSP), Salvador, Brazil; ^15^ Division of Infectious Diseases, Department of Medicine, Vanderbilt University School of Medicine, Nashville, TN, United States

**Keywords:** TB-IRIS, immunologic memory, CD8^+^ T cells, naïve lymphocytes, *M. tuberculosis* infection

## Abstract

**Background:**

Tuberculosis-associated immune reconstitution inflammatory syndrome (TB-IRIS) is a clinical aggravation of TB symptoms observed among a fraction of HIV coinfected patients shortly after the start of antiretroviral therapy (ART). Of note, TB-IRIS is characterized by exacerbated inflammation and tissue damage that occurs in response to the elevated production of CD4^+^ T cell-derived IFN-γ. Nevertheless, the possible participation of CD8^+^ T cells in TB-IRIS development remains unclear.

**Methods:**

We performed a comprehensive assessment of the composition of CD8^+^ T cell memory subsets and their association with circulating inflammation-related molecules in TB-HIV coinfected patients initiating ART.

**Results:**

We found that TB-IRIS individuals display higher frequencies of Antigen-experienced CD8^+^ T cells during the onset of IRIS and that the levels of these cells positively correlate with baseline mycobacterial smear grade. TB-IRIS individuals exhibited higher frequencies of effector memory and lower percentages of naïve CD8^+^ T cells than their Non-IRIS counterparts. In both TB-IRIS and Non-IRIS patients, ART commencement was associated with fewer significant correlations among memory CD8^+^ T cells and cells from other immune compartments. Networks analysis revealed distinct patterns of correlation between each memory subset with inflammatory cytokines suggesting different dynamics of CD8^+^ T cell memory subsets reconstitution. TB-IRIS patients displayed lower levels of memory cells positive for CXCR3 (a chemokine receptor that plays a role in trafficking activated CD8^+^ T cells to the tissues) than Non-IRIS individuals before and after ART. Furthermore, we found that CXCR3^+^ naïve CD8^+^ T cells were inversely associated with the risk of TB-IRIS development. On the other hand, we noticed that the frequencies of CXCR3^+^ effector CD8^+^ T cells were positively associated with the probability of TB-IRIS development.

**Conclusion:**

Our data suggest that TB-IRIS individuals display a distinct profile of memory CD8^+^ T cell subsets reconstitution after ART initiation. Moreover, our data point to a differential association between the frequencies of CXCR3^+^ CD8^+^ T cells and the risk of TB-IRIS development. Collectively, our findings lend insights into the potential role of memory CD8^+^ T cells in TB-IRIS pathophysiology.

## Introduction

*Mycobacterium spp* infection-related diseases remain as major threats to human health, being associated with substantially high rates of morbidity and mortality worldwide, especially in developing countries (1). Noticeably, it is very well documented that humans exhibit a wide range of immune responses against *Mycobacterium tuberculosis* (Mtb) which produces a spectrum of manifestations including latent infection, subclinical disease, and both active pulmonary and extrapulmonary tuberculosis (TB) (2). Interestingly, only a fraction of individuals latently infected with Mtb will ever progress to active TB during the course of their lives. As immunity against Mtb infection occurs in a CD4^+^ T cell-dependent fashion, Human Immunodeficiency Virus (HIV) infection considerably tips the balance towards the mitigation of host protective responses (3). Of note, HIV infection is a major risk factor for active TB development, despite the introduction and overwhelming success of combination antiretroviral therapy (ART) in recent decades (3).

Shortly after the commencement of ART, a fraction of TB-coinfected people with HIV (PWH) may manifest a paradoxical or unmasking worsening of TB symptoms associated with a considerable clinical deterioration in a reaction known as immune reconstitution inflammatory syndrome (IRIS) (4). Clinical features associated with TB-IRIS are diverse and range from mild symptoms (such as fever and lymph node enlargement) to severe respiratory, central nervous system, hematologic, and mesenteric complications, and even death (4). Mechanistically, IRIS occurs when ART-promoted reconstitution of CD4^+^ T cells results in full activation of previously primed Mtb-harboring macrophages. This ultimately leads to an uncoordinated production of pro-inflammatory molecules and considerable inflammation-driven tissue damage (5). Identifiable risk factors for IRIS development include a high pre-ART HIV viral load, advanced immunosuppression associated with severe CD4^+^ lymphopenia, as well as a short interval between antitubercular treatment (ATT) and ART (6).

Several reports suggest that cellular immunity mediated by both innate immune cells and CD4^+^ T cells plays a pivotal role in TB-IRIS immunopathogenesis (7). Previous work from our group demonstrated that hyperactivation of classical (CD14^++^CD16^+^) monocytes before ART is associated with an increased risk of IRIS development (8). Additionally, a comprehensive assessment of functional and memory compartments of CD4^+^ T lymphocytes also revealed distinct patterns of reconstitution during TB-IRIS onset (9). Others have suggested the possible participation of CD8^+^ T cells in the pathogenesis of TB-IRIS, by demonstrating that activation of these cells was associated with robust protective immune responses concomitant to limited immunopathology in the context of TB-IRIS (10). Consistent with this, we have recently identified increased frequencies of cytotoxic Granzyme B-expressing (GzB^+)^ CD8^+^ T cells in TB-IRIS patients that arguably could be a compensatory mechanism in the face of severe CD4^+^ lymphopenia (11). Nevertheless, few reports contemplate the impact of ART-mediated reconstitution of memory CD8^+^ T lymphocyte subsets and whether this could significantly result in an augmented risk for TB-IRIS development.

Here we sought to perform a comprehensive assessment of the memory CD8^+^ T lymphocyte compartment in TB-IRIS. Our findings suggest an enrichment of Antigen-experienced CD8^+^ T cells that positively correlated to Mtb smear grades (or antigen burden) in TB-IRIS patients. Further dissection of the memory compartment revealed that these patients displayed augmented levels of effector memory cells. Of interest, by devising a Spearman correlation-based approach, we noticed differential patterns of correlation between the CD8^+^ T cell memory compartment and surrogate markers of cellular activation and inflammation in our study population. Additionally, the magnitude of naïve CD8^+^ T cell reconstitution after ART was substantially higher among Non-IRIS individuals when contrasted to the TB-IRIS arm of our study. Furthermore, we found that the frequencies of a subpopulation naïve CD8^+^ T cells expressing CXCR3^+^ were independently associated with a decreased probability of TB-IRIS development. Our findings provide insights into the potential role of CD8^+^ T cell memory subset reconstitution in the pathogenesis of TB-IRIS.

## Methods

### Ethics Statement

All clinical investigations were carried out following the principles disclosed in the Declaration of Helsinki. Written informed consent was obtained from all participants before any study procedures. This study was approved by the Scientific Advisory Committee and Institutional Ethics Committee of the National Institute for Research in Tuberculosis (Chennai) and registered on Clinicaltrials.gov (NCT00933790).

### Description of the Study Population

The present study is a retrospective analysis of clinical, laboratory, and microbiologic data collected from a previously published cohort (12). The Indian TB-IRIS cohort study comprised of an observational analysis nested within a randomized controlled trial (NCT00933790) at the National Institute for Research in Tuberculosis (NIRT) in Chennai, India. HIV-1 infected patients with a recent diagnosis of sputum culture-confirmed pulmonary TB were enrolled as previously reported (12). The parent randomized controlled clinical trial primarily compared outcomes of daily versus intermittent anti-TB regimens in the aforementioned group of individuals (13). Eligibility criteria were based on patient age (above 18 years old), confirmation of rifampicin-sensitive TB, and ART-naïve status.

Clinical evaluation and blood samples were collected at pre-ART (baseline) and at the time of IRIS occurrence or equivalent time point (usually between 2 to 6 weeks following ART initiation, hereafter mentioned as timepoint 1). IRIS was diagnosed by following a modified International Network for the study of HIV-associated IRIS (INSHI) guidelines as previously described (12). This took into consideration both expected decreases in HIV plasma viral load (of at least 0.5 logs) and sputum to assess Acid-Fast Bacillus (AFB)-negativity by culture or a decline in Mtb smear grade. The baseline description of patients enrolled in the study is presented in **Supplementary Table 1**.

### Measurement of Plasma Biomarkers

Concentrations of C-reactive protein (CRP) (eBioscience, San Diego, CA), Eotaxin, Fibroblast growth factor (FGF) – basic, FMS-like tyrosine kinase 3 ligand (FLT3L), Granulocyte colony-stimulating factor (G-CSF), intestinal fatty acid binding protein (I-FABP) (Hycult Biotech, The Netherlands), Interferon (IFN)-α, IFN-β, IFN-γ, Interleukin (IL)-1Ra, IL-1β, IL-2, IL-4, IL-5, IL-6, IL-7, IL-8, IL-9, IL-10, IL-12p40, IL-12p70, IL-13, IL-15, IL-17, Interferon-γ-induced protein 10 (IP-10), Monocyte chemoattractant protein (MCP)-1, Macrophage inflammatory protein (MIP)-1, MIP-1α, MIP-1β, Platelet-derived growth factor (PDGF), Regulated on activation normal T cell expressed and secreted (RANTES), soluble CD14 (sCD14), soluble CD163 (sCD163), soluble GzB, soluble Programmed cell death protein (PD) -1, soluble Tissue factor (sTF), Transforming Growth Factor (TGF)-β, Tumor Necrosis Factor (TNF)-α, and vascular endothelial growth factor (VEGF) were assessed using Luminex assay technology (Bio-Plex, Bio-Rad, Hercules, CA) were assessed in cryopreserved plasma samples maintained at −80°C.

### Cell Staining and Flow Cytometry Assays

In order to dissect the immunophenotype of T cells, we evaluated markers associated with activation (HLA-DR), exhaustion (PD-1), proliferation (Ki-67), and cytotoxicity (GzB). Phenotypic identification of memory subsets was based on the expression of CD45 and CCR7 [central memory (CD27^+^CD45RO^+^), naïve (CD27^+^CD45RO^−^), effector (CD27^−^CD45RO^-^), and effector memory (CD27^−^CD45RO^+^]. Functional identification of CD4 ^+^ T cells was based on the expression of CCR6 and CXCR3 receptors [Th1 (CXCR3^+^CCR6^-^), Th2 (CXCR3^-^CCR6^-^), and Th17 (CXCR3^-^CCR6^+^). Briefly, this characterization was conducted by staining aliquots of 250 µL of whole blood on the day of the participant’s visit with the following antibodies: CXCR3, CD3, CD4, CD8, HLA-DR, PD-1, Ki-67, and GzB. All antibodies were obtained from eBioscience (San Diego, CA), Biolegend (San Diego, CA), BD Biosciences (San Jose, CA), and Life Technologies (Carlsbad, CA). The antibody panel was prepared in PBS with 1% BSA for 30 minutes at room temperature. Data were acquired on a BD FACS Canto II flow cytometer (BD Biosciences). All compensation and gate analyses were conducted in FlowJo 9.5.3 (TreeStar, Ashland, OR).

### Network Analysis

The inferential networks were generated from Spearman correlation matrices containing values of each biomarker measured in the plasma samples and flow cytometry markers of T cell activation. All values were inputted and analyzed in the *circusplot* R package. The links shown in the networks represent statistically significant Spearman rank correlations (P<0.05). Additionally, we dissected the structure of networks by calculating the network density. The density measure is defined as follows: density = L/(N (N-1)/2), in which L is the number of observed edges (i.e., Spearman correlations with P<0.05) and N is the total number of the nodes in the network. The density is normalized, ranging between 0 (no edges in the network) and 1 (all possible edges present). Graphics for the network analysis were customized using Adobe Illustrator 21 (Adobe Systems Inc.).

### Data Analysis

Median values with IQR or frequencies of variables were compared using the Mann-Whitney *U* test (when two groups were compared) or the Kruskal-Wallis test with Dunn’s multiple comparisons *ad hoc* analysis (when three groups were compared). Fisher’s exact test or Chi-square tests were used to compare two or three groups, respectively, for proportions. Paired changes from before ART initiation to week 6 or the time of IRIS development were compared using the Wilcoxon matched-paired T-test. Using JMP 10.0 software, geometric mean values (log_10_) for each marker measured at week 0 and week 6 were calculated for the entire study population. To assess the overall pattern of expression of these markers in each clinical group and timepoint, heatmaps were built using variation from the geometric mean value calculated for each candidate biomarker. Unsupervised principal component analysis was performed using the *Factorextra* R package. Unsupervised hierarchical cluster analysis using Ward’s method was employed to reveal patterns of expression in plasma. Receiver operator characteristic (ROC) curve analysis was performed using the pROC package [16]. Throughout the text, a p-value of <0.05 was considered statistically significant after adjustments for multiple comparisons (Holm-Bonferroni’s correction method). The statistical analyses were performed using GraphPad Prism 9.0 (GraphPad Software Inc., USA), the *ggplot2* R package (version 3.10), and STATA 9.0 (StataCorp, TX, USA).

## Results

### TB-Iris Patients Display Higher Frequencies of Antigen-Experienced CD8^+^ T Cells Than Non-Iris Individuals After Art Commencement

**Figure 1:**
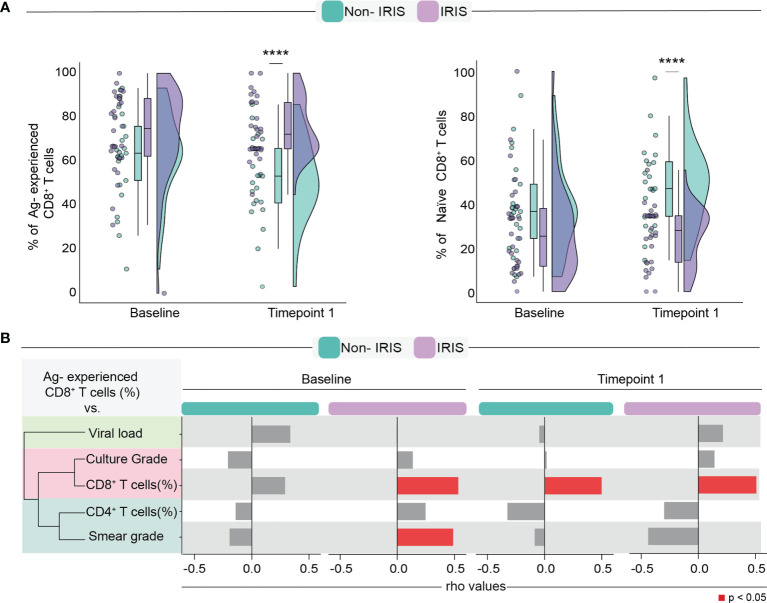
Assessment of Antigen-experienced CD8^+^ T cells before and after ART commencement. **(A)** Comparison of frequencies of Antigen-experienced and naïve CD8 T cells. **(B)** Spearman correlation analysis between Antigen-experienced CD8^+^ T cell frequencies and surrogate clinical parameters at both timepoints. Data were analyzed using the Mann-Whitney test or Wilcoxon matched-pairs test for paired analyses within each study group (****p < 0.0001).

It has been demonstrated that ART-mediated immune reconstitution is associated with a robust increase in CD4^+^ T lymphocyte frequencies in the peripheral blood following treatment initiation (9). However, whether TB co-infection impacts how quickly peripheral memory and naïve lymphocyte counts will rise after ART initiation remains unclear. In addition, little is known about the effects of immune reconstitution in the CD8^+^ T lymphocyte memory compartment, especially in the setting of TB. Here, we sought to investigate whether ART initiation was associated with quantitative changes in the frequencies of naïve and Antigen-experienced (memory) CD8^+^ T cells in individuals who developed TB-IRIS or not. We tested this by employing a flow cytometric approach that considered the expression of CD45RO and CCR7 to identify the following: naïve cells (CD27^+^CD45RO^−^) and Antigen (Ag)-experienced cells (combination of all central memory, effector, and effector memory cells). During IRIS onset, TB-IRIS participants displayed higher percentages of memory and lower percentages of naïve CD8^+^ T cells when contrasted to Non-IRIS individuals (**Figure 1A**). Next, we assessed how Ag-experienced CD8^+^ T cells correlate with several clinical measurements. Of note, we observed that, among IRIS patients, Ag-experienced CD8^+^ T cells positively correlated with Mtb smear grade before ART (**Figure 1B**). Collectively, our data suggest an enrichment of memory CD8^+^ T cells during TB-IRIS onset that correlated with antigen burden.

### TB-IRIS Is Associated With Increased Percentages of Effector Memory CD8^+^ T Cells

**Figure 2:**
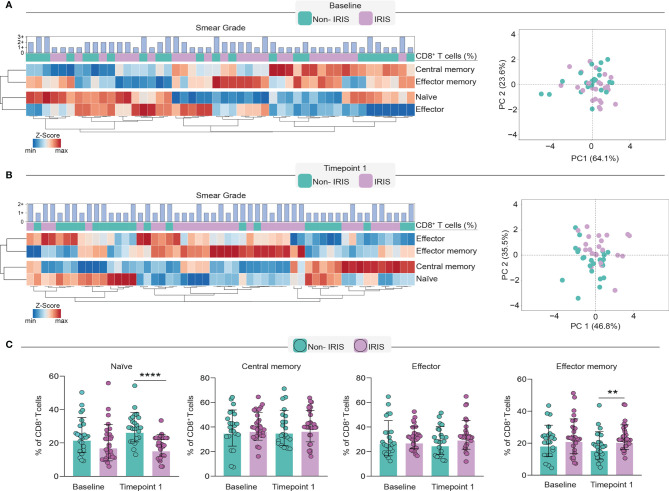
Evaluation of CD8^+^ T cell memory subsets in TB-IRIS pathogenesis. In order to dissect the memory subset of CD8^+^ T cells, we employed a heatmap coupled with hierarchical cluster analysis (Ward’s method) and principal component analysis at baseline **(A)** and timepoint 1 **(B)**. Principal component analysis was performed considering the frequencies of CD8^+^ T cell memory subsets at the aforementioned time points. Scatterplots depict the frequencies of each memory subset within total CD8^+^ T cell counts. **(C)** Comparison of frequencies of memory subsets in TB-IRIS and Non-IRIS patients. Data were analyzed using the Mann-Whitney test or Wilcoxon matched-pairs test for paired analyses within each study group (**p < 0.01, ****p < 0.0001).

Next, we sought to dissect the changes in the CD8^+^ T cell memory compartment during immune reconstitution. To compare differences in memory CD8^+^ T cells, we employed an unsupervised hierarchical cluster analysis to depict the overall profile of each memory lymphocyte subset associated with TB-IRIS or Non-IRIS patients before and after the ART initiation. Additionally, we also applied a principal component analysis (PCA) to assess how efficiently memory CD8^+^ T cell subsets could distinguish our study participants at each timepoint (**Figures 2A, B**). Although our PCA and hierarchical clustering revealed the existence of different profiles of CD8^+^ T cell memory subsets in both timepoints of our study, we noticed a better separation of the patient groups at the time of IRIS occurrence. Of interest, we also noticed that these subsets were also associated with different levels of Mtb smear grade. Particularly, at both timepoints, central memory and naïve subsets were associated with lower Mtb smear grade, while effector cells were associated with higher bacillary loads. Noticeably, while we were not able to observe significant differences in the CD8^+^ T cell memory compartment before ART, we noticed that TB-IRIS patients displayed lower rates of naïve cells and higher rates of effector memory CD8 ^+^ T cells after treatment (**Figure 2C**). Altogether, our data suggest the immunopathogenesis of TB-IRIS could be associated with a predominance of effector memory CD8^+^ T cells and concomitant lower naïve cells after ART in co-infected patients.

### CD8^+^ T Lymphocyte Memory Subsets Correlate With Distinct Profiles of Cellular Activation in Non-Iris And TB-Iris Participants

**Figure 3:**
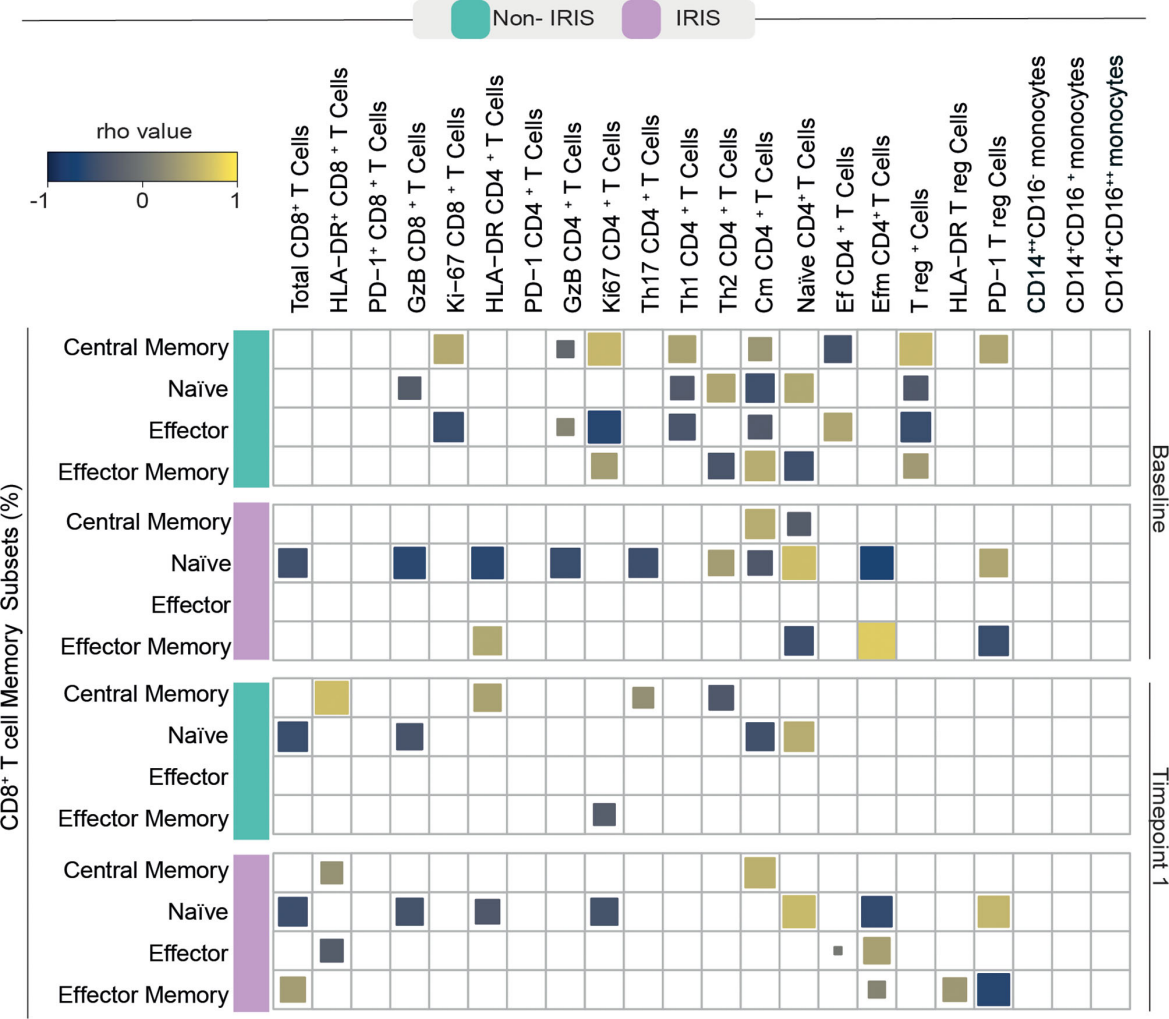
Spearman correlations between CD8^+^ T cell memory subsets and activated adaptive and innate immune cells. A Spearman-based correlation matrix was employed to depict the strength of association among percentages of the memory subsets of CD8^+^ T cells and the frequency of other activated immune cells at Baseline (before ART) and after approximately 2 to 6 weeks following ART initiation (timepoint 1) for Non-IRIS and TB-IRIS subjects. Statistically significant correlations (P < 0.05) after adjustment for multiple measurements are highlighted as yellow squares (positive correlations) or blue squares (negative correlations).

It is well known that immune activation plays a deleterious role in chronic untreated HIV infection which can lead to aberrant reconstitution of the memory lymphocyte compartment and an augmented risk for IRIS development (14). Bearing this in mind, we sought to investigate the patterns of correlation between memory CD8^+^ T cell subsets and other innate and adaptive immune cells in our cohort of TB/HIV co-infected individuals before and after ART commencement. We noticed that, in both studied groups, memory CD8^+^ T cells displayed a high number of correlations with other immune cells before treatment initiation (**Figure 3**).

Before ART, Non-IRIS participants exhibited a higher number of correlations between memory CD8^+^ T cells and other immune cells than their TB-IRIS counterparts. Specifically, Ag-experienced CD8^+^ T cells showed strong correlations with proliferating (Ki-67^+^) CD8^+^ and CD4^+^ T cells, cytotoxic (GzB^+^) CD4^+^ T cells, Th1 and Th2 CD4^+^ T cells, central memory CD4^+^ T cells, as well as total and PD-1^+^ T regulatory (T reg) cells, and effector CD4^+^ T cells.

In the TB-IRIS group, pre-ART frequencies of Ag-experienced CD8^+^ T cells displayed fewer correlations with other immune cells. Ag-experienced cells were correlated with central memory and effector memory CD4^+^ T cells, and PD-1^+^ Tregs. Conversely, naïve CD8^+^ T cells from TB-IRIS patients showed a high number of significant correlations. Naïve CD8^+^ T cells negatively correlated with the total number of CD8^+^ T cells, GzB^+^ CD8^+^ and CD4^+^ lymphocytes, activated and Ki67^+^ CD4^+^ and effector memory CD4^+^ T cells, while positively correlated with the frequencies of naïve CD4^+^ T cells.

After treatment initiation, Non-IRIS individuals exhibited positive correlations between central memory CD8^+^ T cells and PD-1^+^ CD8^+^, activated CD4^+^, and Th17 T cells. In this same group, naïve CD8^+^ T cells were negatively correlated with the total number of CD8^+^ and GzB^+^ T lymphocytes as well as central memory CD4^+^ T cells. Among TB-IRIS individuals, naïve CD8^+^ T cells displayed several negative associations with total frequency of CD8^+^ T cells, GzB^+^ CD8^+^ T cells, activated and proliferating CD4^+^ T cells, and effector memory CD4^+^ T cells. Effector memory CD8^+^ T cells showed strong positive correlations with the total frequency of CD8^+^ T cells, Tregs, and activated Tregs. Conversely, we were able to detect negative correlations between these cells and PD-1^+^ T regs in this same group of patients. In summary, our observations suggest substantial differences in associations among CD8^+^ T cell memory subsets and markers of cellular activation during immune reconstitution in our study population.

### The Magnitude of Memory Subset Variation Differently Correlates With Inflammatory Molecules in Non-Iris And TB-Iris Patients

**Figure 4:**
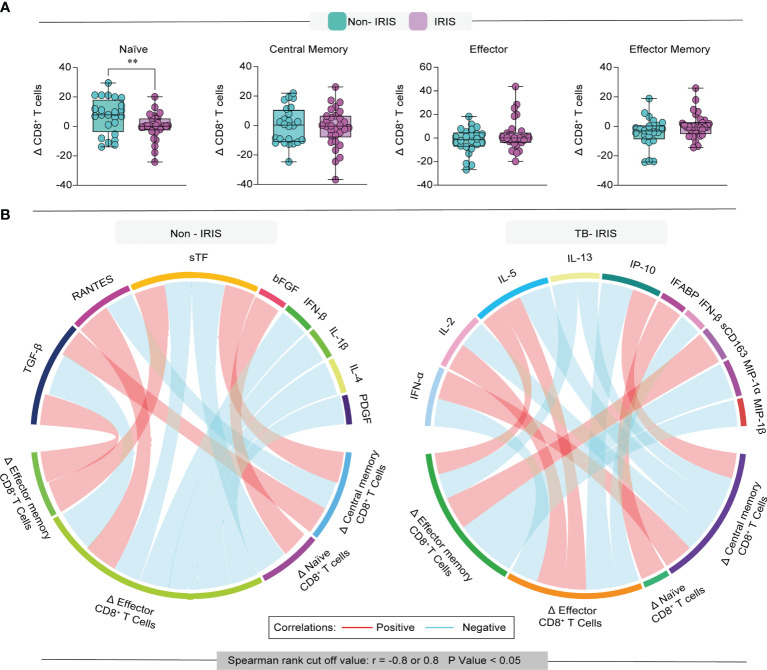
Evaluation of the degree of immune reconstitution in memory CD8^+^ T cells. **(A)** Boxplot depicts the delta (frequency timepoint 1 – frequency baseline) of each memory subset in our study population. **(B)** Network analysis of CD8 T cell memory subsets and association with inflammation-related molecules; correlation matrices were developed with bootstrap (100 ×). Networks depicting only significant correlations (p < 0.05). Data were analyzed using the T-test (**p < 0.01).

Next, we wished to examine the variation of memory CD8^+^ T lymphocyte subsets following treatment initiation in our study population. We found that the degree of variation (Δ [the absolute number of cells at timepoint 1 minus the baseline counts]) of naïve CD8^+^ T cells was higher in Non-IRIS patients compared with TB-IRIS (**Figure 4A**). We could not detect significant variations when examining the other subsets of memory CD8^+^ T cells. These results prompted us to investigate whether the magnitude of this variation of memory subsets correlated with the pre-ART abundance of several inflammatory mediators.

Among Non-IRIS patients, we observed that the Δ of naïve CD8^+^ T cells positively correlated with the level of FGF basic and negatively correlated with soluble Tissue factor (sTF) (**Figure 4B**). Additionally, we observed that Δ of central memory cells displayed negative correlations with RANTES and positive correlations with both TGF-β and sTF. Interestingly, the Δ of effector CD8^+^ T cells from Non-IRIS participants correlated negatively with TGF-β, sTF, IFN-β, IL-1β, IL-4, and PDGF levels, while exhibiting a positive correlation with RANTES. Of note, in this same group, the Δ of effector memory cells correlated positively with sTF and TGF-β.

Concerning TB-IRIS participants, we detected that the Δ of naïve CD8^+^ T cells correlated positively with intestinal fatty acid-binding protein (IFABP). Of note, we noticed that the variation of central memory T cells was positively correlated with IFN-α and IP-10, while negatively associated with IL-2, IL-5, and IL-13. Additionally, we found that the Δ of effector memory cells were correlated with sCD163, MIP-1a, and MIP-1b, whereas the variation of effector cells displayed correlations with IFN-α, IL-2, IL-5, IL-13, IP-10, and IFN-β.

Collectively, our observations suggest that the magnitude of memory CD8^+^ T lymphocyte subset reconstitution displays different correlation patterns with inflammatory mediators in TB-IRIS patients, which may have implications for the pathology of TB-IRIS.

### Non-Iris Patients Display Sustained Higher Frequencies of CXCR3^+^CD8^+^ T Cells Than Their TB-Iris Counterparts

**Figure 5:**
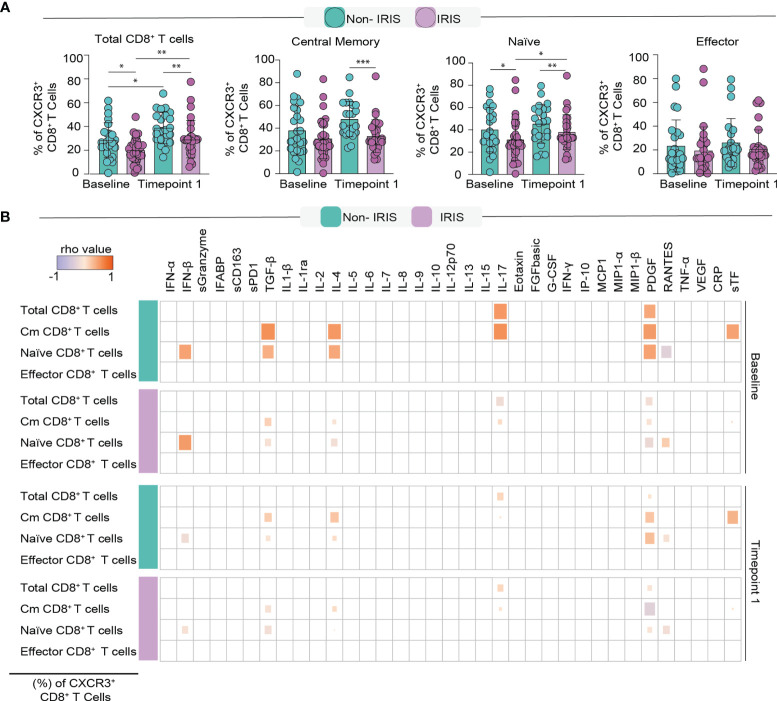
Assessment of CXCR3^+^ memory CD8^+^ T cells in TB-IRIS and Non-IRIS individuals. **(A)** Frequencies of CXCR3^+^ in each memory compartment of CD8^+^ T cells. **(B)** A Spearman-based correlation matrix was employed to depict the strength of association among percentages of the memory subsets of CD8^+^ T cells and the concentration of pre-ART inflammatory molecules in Non-IRIS and TB-IRIS subjects. Statistically significant correlations (P < 0.05) after adjustment for multiple measurements are highlighted as orange squares (positive correlations) or purple squares (negative correlations). *p < 0.05, **p < 0.01, ***p < 0.001.

We next ascertained whether the distinct composition of the CD8^+^ T cell memory compartment seen between TB-IRIS and Non-IRIS groups was related to a differential commitment to an effector fate during IRIS onset. To evaluate this possibility, we employed a flow cytometric approach to measuring the expression of CXCR3 ^+^ in the memory subsets of CD8^+^ T cells. Of note, CXCR3 is a chemokine receptor that plays a role in trafficking activated CD8^+^ T cells to peripheral tissues (15). We observed that Non-IRIS individuals displayed higher levels of total CD8^+^ T cells expressing CXCR3^+^ before and after ART commencement (**Figure 5A**). A similar pattern of CXCR3^+^ expression was seen in naïve CD8^+^ T cells. Furthermore, the start of ART was associated with an increase in CXCR3^+^ expression in both groups. Interestingly, we also noted higher frequencies of CXCR3^+^ central memory CD8^+^ T cells in Non-IRIS patients than in TB-IRIS patients.

Subsequently, we sought to correlate the expression of CXCR3^+^ memory subsets with circulating levels of several cytokines and chemokines (**Figure 5B**). Preceding ART, CXCR3^+^ central memory cells displayed strong positive correlations with TGF-β, IL-4, IL-17, PDGF, and sTF levels in Non-IRIS individuals. This same correlation pattern was seen in TB-IRIS; however, the magnitudes of the Spearman correlations were considerably lower. Of interest, the number of CXCR3^+^ naïve T cells correlated positively with IFN-β, TGF-β, IL-4, and PDGF levels, while correlating negatively with RANTES. We could not detect any significant correlation between CXCR3^+^ effector T cells and the measured inflammatory molecules. Conspicuously, after the start of ART, the number and magnitude of correlations among CXCR3^+^ memory T cells and inflammatory mediators were considerably reduced in both groups. Particularly, we noticed that the number of CXCR3^+^ naïve T cells negatively correlated with sGzB and TNF-α levels in Non-IRIS patients. Among TB-IRIS patients, CXCR3^+^ central memory cells negatively correlated with PDGF levels. Together, these findings suggest strong associations between CXCR3^+^ memory CD8^+^ T cells and inflammation-related biomarkers, which could comprise important immunologic networks that play a crucial role in TB-IRIS development.

We also wished to evaluate whether delayed ART following ATT initiation could impact the frequencies of CXCR3^+^ memory CD8^+^ T cells. We noted that, in the entire study population, time to ART was positively correlated with pre-ART frequencies of naïve and central memory CD8^+^ T cells expressing CXCR3 (**Supplementary Figure 1**). Remarkably, time to ART was positively correlated with the frequency of CXCR3^+^ central memory CD8+ T cells in the Non-IRIS group at timepoint 1. Additionally, we also observed that patients who initiated ART more than 4 weeks after ATT initiation showed augmented frequencies of all memory CD8^+^ subsets expressing CXCR3 after up at timepoint 1 (**Supplementary Figure 1**). In summary, our results highlight that delayed ART initiation is associated with an enrichment of CXCR3^+^ CD8^+^ T cells with possible implications in TB-IRIS pathogenesis.

### The Frequencies of CXCR3^+^ Memory CD8^+^ T Cell Subsets Are Differently Associated With the Risk of TB-Iris Development

**Figure 6:**
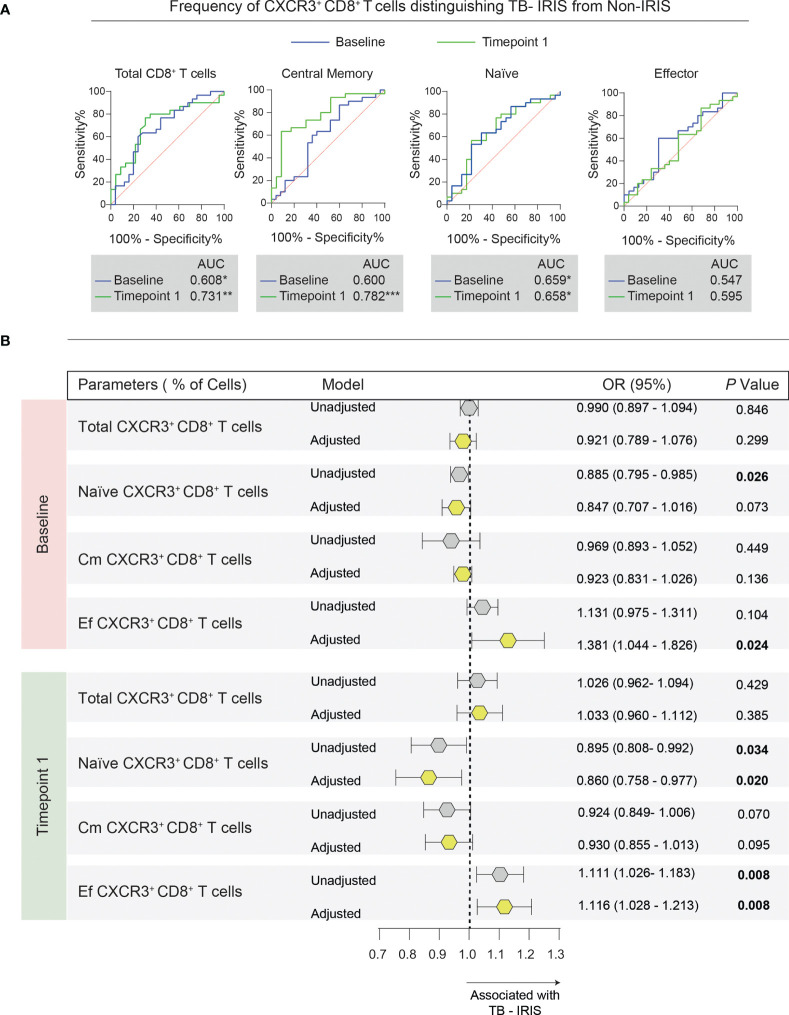
Association between memory CD8^+^ T cell subsets expressing CXCR3 and the risk of TB-IRIS development. **(A)** Receiver operator characteristic (ROC) curves were employed to discriminate TB-IRIS from Non-IRIS patients at both timepoints taking into account the frequency of each flow cytometric population. **(B)** Multivariate regression analysis to evaluate the association between the frequencies of CXCR3^+^ memory CD8^+^ T cells and *Mycobacterium tuberculosis* immune reconstitution inflammatory syndrome (TB-IRIS). The multivariate model included the following variables: CD4^+^ T cell counts, viral load, smear grade, and the number of days between ATT and ART initiation. Odds ratios (OR) represent the 95% confidence interval increase in the frequency of each lymphocyte population. P values are shown in bold. Gray polygons represent unadjusted models, while yellow ones represent adjusted models.

Next, we broadened the scope of our analysis to evaluate whether the frequency of CXCR3^+^ memory CD8^+^ T lymphocyte subsets could predict and/or diagnose TB-IRIS in our study population. We first employed ROC curve analysis to assess the power of discrimination based on CXCR3^+^ expression at baseline and timepoint 1 (approximately 2 to 6 weeks after ART start) (**Figure 6A**). Of note, we found that the total frequency of CXCR3^+^ CD8^+^ T cells could discriminate TB-IRIS from Non-IRIS subjects at both timepoints. Importantly, our ROC curves analysis obtained high AUC values for CXCR3^+^ central memory cell frequency at timepoint 1, further highlighting its potential diagnostic potential. Additionally, the frequencies of naïve CD8^+^ T cells expressing CXCR3^+^ were also statistically significant at distinguishing our patients at both timepoints.

Next, we applied a multivariate regression model using the pre-ART frequencies of CXCR3^+^ memory CD8^+^ T cells to predict TB-IRIS (**Figure 6B**). After adjustment for pre-ART CD4^+^ T cell counts, viral load, smear grade, and the time between ATT and ART, we found that baseline frequencies of CXCR3^+^ naïve CD8^+^ T cells were independently associated with a lower odds ratio of TB-IRIS development (OR, 0.885 [95% CI (0.795 – 0.985)]). On the other hand, the pre-ART percentages of CXCR3^+^ effector CD8^+^ T cells were associated with a higher risk of TB-IRIS (OR, 1.381 [95% CI (1.044 – 1.826)]).

Additionally, by applying the same model to the frequencies of CXCR3^+^ memory CD8^+^ T cells at timepoint 1, we noted that increased frequencies of CXCR3^+^ naïve CD8^+^ T cells were associated with reduced risk of TB-IRIS development (OR, 0.860 [95% CI (0.758 – 0.976)]). Conversely, we found that higher percentages of CXCR3^+^ effector CD8^+^ T cells were independently associated with a higher risk of TB-IRIS (OR, 1.116 [95% CI (1.028 – 1.213)]). Collectively, our data suggest that the abundance of CXCR3^+^ subpopulations of naïve and memory CD8^+^ T cells could be employed to assess the risk of TB-IRIS development.

## Discussion

In our current investigation, we sought to examine the alterations in the CD8^+^ T lymphocyte memory compartment in the face of ART-mediated immune reconstitution in highly immunosuppressed TB-HIV coinfected individuals. Here, we report an inverse association between the frequencies of naïve CXCR3^+^ CD8^+^ T cells and the probability of TB-associated IRIS occurrence. Conversely, we also detected that the frequencies of the CXCR3^+^ effector CD8^+^ T cell subset were associated with an increased risk of TB-IRIS. There is robust evidence on the contributions of CD8^+^ T cells in the mobilization of appropriate immune responses in several infectious diseases, including HIV infection and TB (16–18). A previous study by our group addressed the nuances of T lymphocyte activation in TB-IRIS development and outlined the participation of cytotoxic CD8^+^ T cells during ART-promoted immune reconstitution (11).

It has been demonstrated that immunological memory, a distinguishing feature of the immune system in eliciting more robust responses upon antigen re-encounter, plays a role in the immunopathogenesis of several diseases and vaccination (19–22). Our working hypothesis is that TB-IRIS patients experience an aberrant reconstitution of CD8^+^ T cell memory subsets after ART, which arguably could contribute to the amplification of uncontrolled lung inflammation and tissue damage. We noticed that TB-IRIS patients displayed a higher prevalence of these cells that were correlated with higher Mtb bacillary burden before ART and these individuals also exhibited lower rates of naïve CD8^+^ T cells. These findings are in agreement with those previously found by our group when investigating the memory compartment of CD4^+^ T cells (9). More specifically, our findings suggest an enrichment of effector memory CD8^+^ T cells in TB-IRIS patients during the occurrence of IRIS, which may represent the presence of clonally expanded CD8^+^ T cells that are specific for Mtb antigens. Further studies to assess whether these populations of effector memory CD8^+^ T cells exhibit a more cytotoxic phenotype are warranted.

Our group has previously demonstrated that pre-ART cellular activation predisposes individuals to TB-IRIS (8, 11). Notably, TB-IRIS and Non-IRIS patients differed substantially in the number of correlations found before ART initiation. This could be explained by CD8^+^ T cells playing a pivotal role in orchestrating different immunologic responses in these distinct settings of severe immunosuppression. Likewise, this could be interpreted as a compensatory mechanism in which CD8^+^ memory T cells take a central stage in maintaining homeostasis during the processes of immune reconstitution. Particularly, central memory T cells correlated positively with cycling CD4^+^ and CD8^+^ T cells as well as total and exhausted Tregs among Non-IRIS individuals. Conversely, at timepoint 1, TB-IRIS patients displayed more significant correlations, especially with memory CD4^+^ T cells and T regs.

Spearman correlation analysis revealed that the magnitude of memory subset variation between TB-IRIS and Non-IRIS patients displayed different correlational patterns with inflammatory mediators. On one hand, we find that the immune reconstitution of memory T cell compartments in the Non-IRIS group was more heterogeneous, displaying correlations with the pre-ART abundance of both anti- and pro-inflammatory molecules (especially TGF- β, IL-4, sTF, and IFN-β). On the other hand, among TB-IRIS the variation in memory T cell subsets exhibited a higher number of correlations with inflammatory mediators. These results seem in good agreement with previous reports of increases of Th1 inflammatory mediators (namely IL-2, IFNs, TNF-α) in TB-IRIS (23, 24). One could argue that aberrant reconstitution of memory CD8^+^ T lymphocyte compartment in the face of persistently high bacillary burden is coupled with both detrimental inflammation and clinical deterioration seen in TB-IRIS patients.

We also wished to ascribe whether the observed heterogeneity in memory compartment reconstitution between our study groups was due to *de novo* differentiation of naïve T cells in the face of CD4^+^ T cell rise or to a redistribution of previously differentiated memory CD8^+^ T cells from lymphoid tissues to the hematogenous compartment. Regarding the quantitative recovery of CD4^+^ T cells, it has been to occur in two main phases. The first phase, which happens from 1 to up to 6 months after treatment initiation, is characterized by the redistribution of memory T cells that were trapped in lymphoid organs to the periphery, coupled with a substantial reduction in viral load, whereas the other phase can last many years and is characterized by a slow and gradual increase of CD4^+^ cells (25). Along these lines, it has been suggested that the levels of CD8^+^ T cells also rise in the circulation after treatment due to non-specific redistribution (23). Our analysis of CD8^+^ T cells expressing CXCR3^+^, a chemokine receptor largely recognized for directing cells to sites of inflammation, revealed that Non-IRIS patients display higher rates of CXCR3^+^ cells in the periphery before and after treatment. One could speculate that the lower degree of CXCR3^+^ circulating CD8^+^ T cells among TB-IRIS patients reflects retention of these cells in persistently inflamed lymphoid tissues. More specifically, one could hypothesize that Mtb- harboring cells, more predominant in TB-IRIS patients at IRIS onset, could produce higher amounts of CXCR3^+^ ligands (ie. CXCL9, C XCL10, CXCL-11), therefore increasing the trafficking of CXCR3^+^ CD8^+^ T cells into sites of inflammation. The abundance of CXCR3^+^ ligands in the settings of TB-IRIS and the identification of cell populations that secrete them are warranted to be conducted.

The limitations of our study include the small number of patients included in our study cohort with a large number of comparisons and the lack of functional assays to assess cells. Another important limitation is the lack of tissue, to evaluate T-cells in areas of infection and inflammation. The strengths of our study include the selection of a well-described cohort of patients who were not previously treated for either HIV infection or TB, had close clinical follow up along with detailed microbiologic evaluation and same day flow cytometric analysis before the administration of anti-inflammatory medications at the IRIS timepoint. Taken together, our findings provide significant insight into the role of immunological memory of CD8^+^ T cells in the immunopathogenesis of TB-IRIS.

## Data Availability Statement

The raw data supporting the conclusions of this article will be made available by the authors, without undue reservation.

## Ethics Statement

The studies involving human participants were reviewed and approved by Scientific Advisory Committee and Institutional Ethics Committee of the National Institute for Research in Tuberculosis (Chennai) and registered on Clinicaltrials.gov (NCT00933790). The patients/participants provided their written informed consent to participate in this study.

## Author Contributions

BA, IS, SS, BP, and AS conceptualized the study. BA and IS supervised the immunological study. GN and SS supervised the clinical study. IS, SS, and AS acquired funding for research. GN, RS, KS, BP, IS, and BA performed the clinical assessments. SA, KN, KA, BA, and LA performed the experiments. RT, BB-D, MA-P, and AQ analyzed the data. RT and BA drafted the first version of the manuscript. All the authors revised the manuscript.

## Funding

This work received support from the Intramural Research Program of the National Institute of Allergy and Infectious Diseases (NIAID/NIH) and by the Intramural-to-India grant from the US-India Co-operative research program. This study was also financed in part by Coordenação de Aperfeiçoamento de Pessoal de Nível Superior (CAPES) (Finance Code 001). The work of BA is supported by the Intramural Research Program of the Oswaldo Cruz Foundation (FIOCRUZ) and the National Council for Scientific and Technological Development (CNPq), Brazil. RT, MA-P and BB-D were supported by Ph.D. fellowships from CAPES.

## Conflict of Interest

The authors declare that the research was conducted in the absence of any commercial or financial relationships that could be construed as a potential conflict of interest.

## Publisher’s Note

All claims expressed in this article are solely those of the authors and do not necessarily represent those of their affiliated organizations, or those of the publisher, the editors and the reviewers. Any product that may be evaluated in this article, or claim that may be made by its manufacturer, is not guaranteed or endorsed by the publisher.

## Supplementary Material

Supplementary Figure 1 Expression of CXCR3^+^ in memory CD8^+^ T cells from patients that initiated ART with more or less than 4 weeks difference from ATT initiation. We employed a hierarchical cluster analysis of z-score normalized values of CXCR3^+^ memory CD8^+^ T lymphocytes at Baseline **(A)** and at timepoint 1 **(B)**. The right panel depicts Spearman correlation coefficient values between time from ATT to ART initiation vs. the frequency of indicated parameters. **(C)** Violin plots represent the frequencies of each CXCR3^+^ memory CD8^+^ T cell in our study groups. Data were analyzed using the Mann-Whitney test or Wilcoxon matched-pairs test for paired analyses within each study group (**p < 0.01, ****p < 0.0001). Data were analyzed using the Mann-Whitney test or Wilcoxon matched-pairs test for paired analyses within each study group (* p < 0.05, **p < 0.01, ***p < 0.001, ****p < 0.0001).

Supplementary Table 1 Characteristics of the study participants.

Supplementary Table 2 Antibody Panel used to assess CD8^+^ T lymphocyte memory subset.
