# Supplementary material for: Frequency of CXCR3+ CD8+ T-Lymphocyte Subsets in Peripheral Blood Is Associated With the Risk of Paradoxical Tuberculosis-Associated Immune Reconstitution Inflammatory Syndrome Development in Advanced HIV Disease
Source: Front Immunol. 2022 Apr 1;13:873985. doi: 10.3389/fimmu.2022.873985 (PMC9011055; doi:10.3389/fimmu.2022.873985)
Supplement: Supplementary Figure 1 — Expression of CXCR3+ in memory CD8+ T cells from patients that initiated ART with more or less than 4 weeks difference from ATT initiation. We employed a hierarchical cluster analysis of z-score normalized values of CXCR3+ memory CD8+ T lymphocytes at Baseline (A) and at timepoint 1 (B). The right panel depicts Spearman correlation coefficient values between time from ATT to ART initiation vs. the frequency of indicated parameters. (C) Violin plots represent the frequencies of each CXCR3+ memory CD8+ T cell in our study groups. Data were analyzed using the Mann-Whitney test or Wilcoxon matched-pairs test for paired analyses within each study group (**p < 0.01, ****p < 0.0001). Data were analyzed using the Mann-Whitney test or Wilcoxon matched-pairs test for paired analyses within each study group (* p < 0.05, **p < 0.01, ***p < 0.001, ****p < 0.0001). [file DataSheet_1.pdf]

Supplementary Table 1. Characteristics of the study participants

Patient characteristics	Non Tb IRIS (N =25)	TB-IRIS (N= 31)	p-value
**Male Gender - n (%)**	20 (80%)	18 (58%)	0.735
**Median Age - years (IQR)**	37 (31-40)	36 (27-46)	0.967
**Median BMI -Kg/m^2 (IQR)**	40.5 (35.7-46.5)	42.0 (36.0-48.0)	0.732
**Median Time to ART - days (IQR)**	43 (23-68)	20 (14-30)	**< 0.001**
**Hematologic characteristics**			
**$CD4^+$ T lymphocytes/µL – median (IQR)**	156 (89-264)	93 (39- 135)	**0.005**
**$CD8^+$ T lymphocytes/µL – median (IQR)**	459 (297 -727)	765 (311 -1095)	0.109
**HIV RNA copies – median log_{10}/mL plasma (IQR)**	5.3 (4.5 -5.6)	5.9 (5.4 – 5.9)	**< 0.001**

Table Note: Data represent no (%) of participants unless otherwise indicated. Data were compared using the Mann Whitney U test (continuous variables) or the Fisher's exact test. IQR=interquartile range; BMI=body mass index; ART=antiretroviral therapy

Supplementary Table 2. Antibody Panel

Marker	Fluorochrome	Clone	Manufactor	Catalogue #
CD3	APC-Cy™7	SK7	BD pharmigen	557832
CD4	Pacific Blue	RM4-5	ebioscience	MCD0428TR
CD8	Qdot 605	3B5	Invitrogen	Q10009
CD45RO	Pe-Cy5	UCHL1	ebioscience	15-0457-73
CD27	FITC	M-T271	BD pharmigen	555440
CCR6	Pe	11A9	BD pharmigen	559562
CXCR3	APC	1C6/CXCR3	BD pharmigen	550967

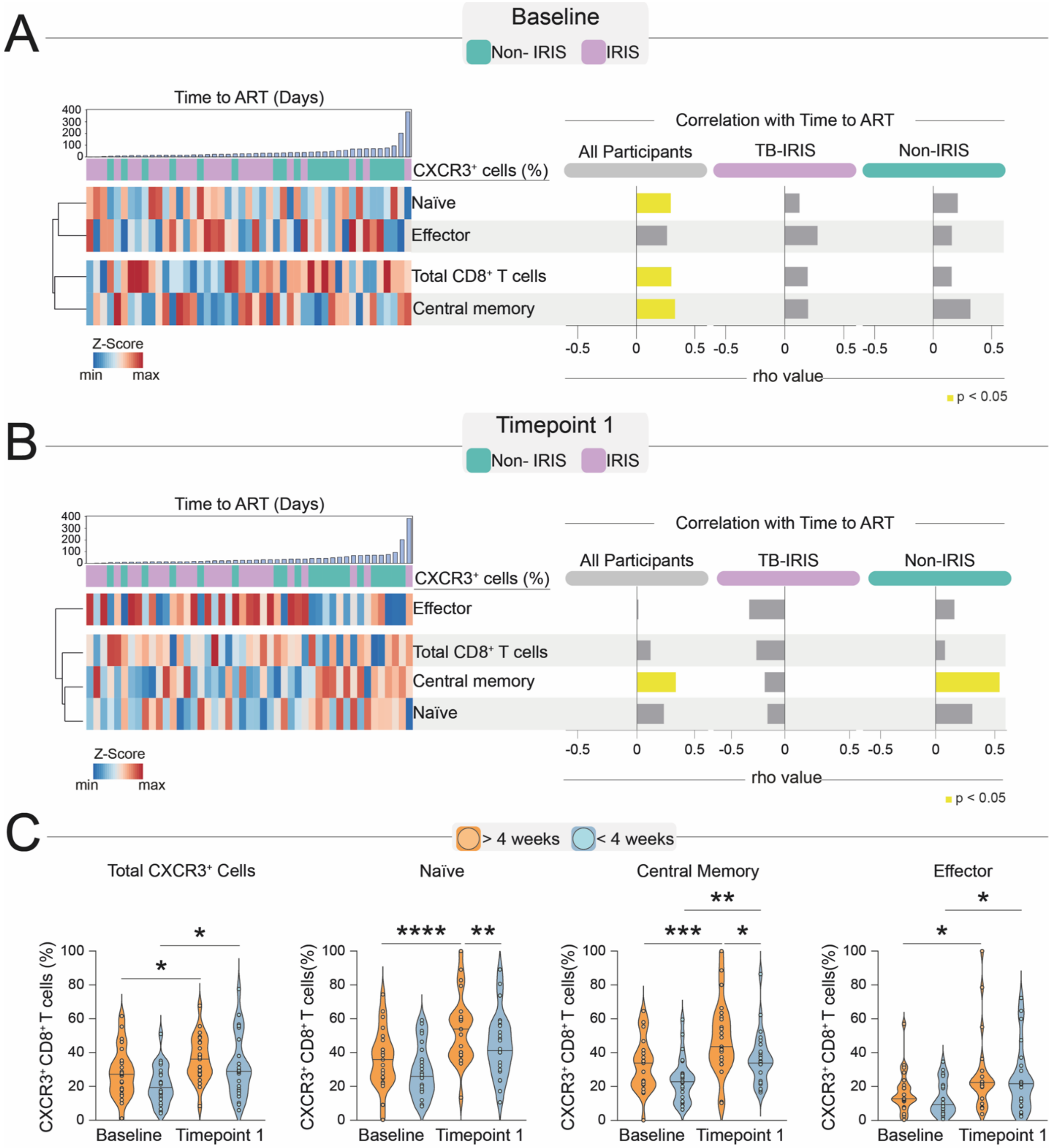

Supplementary Figure 1 - Expression of CXCR3⁺ in memory CD8⁺ T cells from patients that initiated ART with more or less than 4 weeks difference from ATT initiation. We employed a hierarchical cluster analysis of z-score normalized values of $CXCR3^+$ memory $CD8^+$ T lymphocytes at Baseline (A) and at timepoint 1 (B). The right panel depicts Spearman correlation coefficient values between time from ATT to ART initiation vs. the frequency of indicated parameters. (C) Violin plots represent the frequencies of each $CXCR3^+$ memory $CD8^+$ T cell in our study groups. Data were analyzed using the Mann-Whitney test or Wilcoxon matched-pairs test for paired analyses within each study group (**$p < 0.01$, ****$p < 0.0001$).
